# Bibliometric Analysis of Studies on Whole Body Electromyostimulation

**DOI:** 10.3390/biology11081205

**Published:** 2022-08-12

**Authors:** Luiz Rodrigues-Santana, José Carmelo Adsuar, Ángel Denche-Zamorano, Alejandro Vega-Muñoz, Guido Salazar-Sepúlveda, Nicolás Contreras-Barraza, Carmen Galán-Arroyo, Hugo Louro

**Affiliations:** 1 Faculty of Sport Sciences, University of Extremadura, 1003 Cáceres, Spain; 2 Promoting a Healthy Society [PHeSo], Research Group, Faculty of Sport Sciences, University of Extremadura, 1003 Cáceres, Spain; 3 Public Policy Observatory, Universidad Autónoma de Chile, Santiago 7500912, Chile; 4 Departamento de Ingeniería Industrial, Facultad de Ingeniería, Universidad Católica de la Santísima Concepción, Concepción 4090541, Chile; 5 Facultad de Economía y Negocios, Universidad Andres Bello, Viña del Mar 2531015, Chile; 6 Sport Sciences School of Rio Maior, Research Center in Sport Science, Health and Human Development, 2040-413 Rio Maior, Portugal; 7 Life Quality Research Center, 5000-801 Vila Real, Portugal

**Keywords:** WB-EMS, sports health, fitness, exercise, strength, bibliometrics

## Abstract

**Simple Summary:**

This work consists of a review with bibliometric analysis on WB-EMS. One hundred and two articles were analyzed in the period from 2010 to 2022 to find out which country, author and institutions produce the most knowledge on this topic. The results of this work are important to know to determine publication growth trend as well as the most relevant clusters and authors.

**Abstract:**

Whole Body Electromyostimulation [WB-EMS] is a training methodology that applies electrostimulation in the main muscle groups of the human body superimposed with active training exercises. This study aims to carry out a bibliometric analysis on WB-EMS to provide an overview of the state of research and provide new insights for research in the field. Method: One hundred and two citations extracted were examined using a bibliometric approach based on data stored in the Web of Science Core Collection, applying traditional bibliometric laws, and using VOSviewer and excel for data and metadata processing. Results: Among the results, this study points out that Germany is the country that produces more scientific knowledge on WB-EMS. Wolfgang Kemmler is the most relevant author in this field. Moreover, Frontier of Physiology is the journal where the authors publish the most. Conclusion: Research on WB-EMS has been growing in recent years. German and Spanish researchers lead two clusters where most studies and collaborations in this field are carried out. These findings will provide a better understanding of the state of WB-EMS research and may guide the emergence of new lines of investigation and research ideas.

## 1. Introduction

This work aimed to analyze the interest and scientific evolution of Whole Body Electromyostimulation (Wb-EMS) training though a bibliometric analysis. WB-EMS emerged about a decade ago as an alternative to conventional training [1]. This training methodology uses technology combined with physical exercise. Through a special suit with electrodes, electrical stimulus (electromyostimulation) is sent to the main muscles of the human body. The number of electrodes varies according to the devices and brands (from six to ten pairs) and stimulation of the quadriceps, hamstrings, glutes, dorsal, pectoral, abdominal, biceps and triceps is common [2].

Considered by some authors as a time-efficient and safe methodology for those who do not want or do not like conventional methodologies [3], WB-EMS has gained more and more space and more practitioners worldwide. It is estimated that there will be over 7000 WB-EMS studios only in Europe with hundreds of thousands of practitioners.

Although this technology has been around for a long time, the number of scientific investigations is insufficient and unclear about its effects and benefits. Moreover, with a more detailed analysis of the fields on the subject, we can find three main applications of WB-EMS: Performance [4,5,6], Rehabilitation [7] and Health and Wellness [8].

Regarding the population, the WB-EMS has been studied and applied to the elderly [2], special populations that suffer from comorbidities [9,10,11,12] (e.g., obesity, cancer and sarcopenic) and also to athletes [13,14].

### 1.1. Effects of Whole Body Electromyostimulation on the Human Body

Although the use of WB-EMS is more than a decade old, the use of muscular electrostimulation (in an isolated and localized form) is thousands of years old. The earliest known use was by the Egyptians over 2000 years ago, who discovered the electrical properties in fish and used them to treat some diseases. From the 1970s onwards its use became more popular for physiotherapy, strengthening and fitness.

In the most recent use of this methodology in a global way (whole body), activity (combined with exercise) is based on electrical stimulation superimposed onto voluntary contraction. In context of chronic application, theoretically, the superimposition of electrical stimulation onto voluntary contraction recruits additional muscle fibers, producing more force, potentiating physiological adaptations and causing improvements in muscle power, strength or endurance [15].

The literature shows us some of these effects caused by WB-EMS training. In some randomized clinical trials, the experimental group significantly improved their strength and muscle mass [16,17]. Loss of fat mass and changes in body composition are also other effects demonstrated in some studies [18,19,20]. On the other hand, there are also studies that report improvement in chronic low back pain [21,22,23] and improving sports performance by increasing jumping, sprinting and muscle power [24,25,26].

### 1.2. Type of Current and Training Protocols Used

Most studios use the same type of current: bipolar, squared, with 85 Hertz, 350 milliseconds of pulse width and a 4 s contraction time [2,9,16,20,27]. The intensity of the current is, in most cases, controlled by the Borg subjective perception scale (RPE) [18,28,29]. Recently, some authors have used the maximum stimulus tolerance to define the intensity of the current [30,31]. The total time of sessions is up to 20 min in the most relevant studies. Moreover, there are few studies carried out with training with WB-EMS longer than 20 min.

The most cited articles in the literature show us that the exercise protocols performed with WB-EMS are mostly strength exercises with body weight or with very little additional load [32,33,34,35,36]. Considering the short duration of this training methodology, 6–10 exercises are performed in 1–2 sets of 8–12 repetitions in most randomized clinical trials of a recent metanalysis [37]. Power exercise protocols [5,38] were also used in some studios, as well as strength and hypertrophy exercises [39].

When it comes to cardiovascular endurance in the few studies where these types of exercises have been done, they are either done in metabolic circuits (e.g., high-intensity interval training) or are combined with strength exercises in a two-part hybrid training session [25,40,41].

Thus, considering the short time of existing research with WB-EMS and the increase of this practice worldwide, it is necessary to carry out a wide and updated meta-analytic study that provides an overview to the scientific, training and practitioner communities, which is feasible through a bibliometric approach that analyzes data and metadata from pre-existing specialist articles.

Therefore, the first aim of this study was to identify which authors, countries and journals publish the most on Whole Body Electromyostimulation. Secondly, we must find out if there is an exponential growth of publications over the years on this topic through a bibliometric analysis.

## 2. Materials and Methods

For the design of this study, a search was carried out in the main collection of the Web of Science (WoS), considering all articles published in journals indexed by WoS in the Science Citation Index (WoS-SCI-E) and Social Science Citation Index [42], based on a search vector referring to WB-EMS (Topic = ((Whole AND body) NEAR/0 electromyo*) or ((Whole AND body) NEAR/0 electrosti*) and Articles or Review Articles (document types), without restricting temporal parameters. We only extracted articles from WoS journal indexing database, since many studies [43,44,45,46,47] have revealed that the results of systematic reviews may vary according to the database used once different criteria to calculate the impact factor of journals can be used [46,47]. This procedure was performed on 30 April 2022. The data set was filtered, correcting duplications of authors and affiliations.

A general bibliometric analysis of the article set obtained was carried out, analyzing the scientific production referring to the field of research of this study, as well as the scientific trends in this field, checking if these follow an exponential growth. According to “Price’s Law on Exponential Science Growth” [48,49] the annual exponential growth of publications on a topic indicates that there is a strong interest among the scientific community, with a large critical mass of researchers developing this area of knowledge. The concentration of publications by journals was evaluated by applying Bradford’s law of concentrations, distributing these in terciles, according to the number of articles published in them, and obtaining the core of journals in which at least 33% of the total publications are concentrated, ratio *n*0 (journals in the core): *n*1 (journals in the zone 1): *n*2 (journals in zone 2), being “*n*”, Bradford multiplier, the average growth rate in the number of journals from one zone to the next [50,51]. In any field of knowledge, most of the articles come from a small portion of prolific authors. Lotka’s law was applied to identify the prolific authors so that they could be studied in isolation [51,52]. The most prominent articles and authors were identified by applying the Hirsch index (h-index), thus considering the “*n*” articles/authors cited at least “*n*” times or more [53]. Finally, the most frequently used keywords in the article set were highlighted by applying Zipf’s law of words [54]. VOSviewer software was used to perform the processing and visualization of the dataset, as well as co-citation and co-occurrence, performing fragmentation analysis with visualization outputs of temporal trends [55,56].

## 3. Results

**Figure 1:**
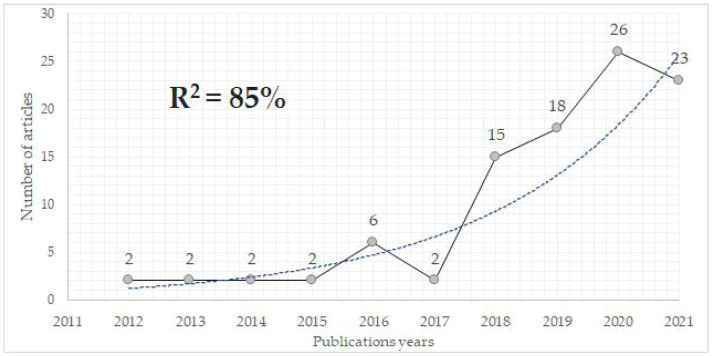
Temporary publication trend on WB-EMS between 2011 and 2021. Gris line: number of articles published per year. Blue line: exponential growth trend.

A total of 102 papers (85 articles and 17 Review articles) were published between 2010 and 2022, included in 30 WoS categories, with 50% of the publications concentrated in two categories: Physiology (28 articles) and Sport Sciences (23 articles). Until 2012, no annual publication continuity was found, and no articles were published in 2011, so the analysis of the exponential growth of annual publications was performed between 2012 and 2021, the last complete year given the impossibility of performing this analysis with values equal to zero in any of the years. It was found that the publications adjusted to an exponential growth curve (R^2^ = 85%) for the period analyzed (Figure 1). The time period in which the newest median number of publications (contemporaneous articles) was concentrated was from 2020 to 2022 (51 articles), with the oldest median being concentrated between 2010 and 2018 (51 articles), meaning that it is a recently expanding topic.

Applying Bradford’s law of dispersion of the literature, three levels of concentration of publications were established, ratio: 1: *n*: *n*2 (% error = −4.3). Of the publications, 34% (35 articles) were concentrated in three journals, forming the core of publication: *Frontiers in Physiology* (23 articles), *Clinical Interventions in Aging* (6 articles) and *International Journal of Environmental Research and Public Health* (6 articles). Publication Zone 1 was made up of 10 journals, concentrating 23% of the publications. These journals presented between two and three articles. Zone 2 was made up of 44 journals, concentrating 43% of the publications (44 articles); one article per journal. Table 1 shows the journals that conformed the Bradford Core + Zone 1.

**Figure 2:**
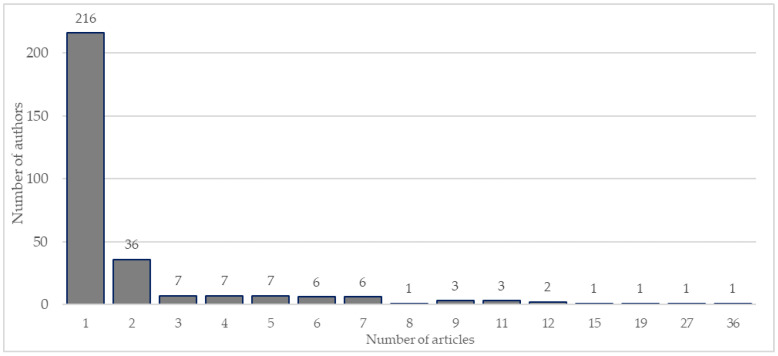
Histogram of publications per author on WB-EMS.

A total of 298 authors were found in the 102 articles included. Of the authors, 75% (216) had published only one article, compared to 11% (32 authors) with two articles. The other authors published between 3 and 36 articles (Figure 2).

According to Lotka’s law, it was estimated that the prolific authors should be the 17 with the highest number of publications (square root of 298). Then, 19 authors with the most published articles were considered the prolific authors: 13 authors with 8 or more publications + 6 authors with seven publications (Table 2).

**Figure 3:**
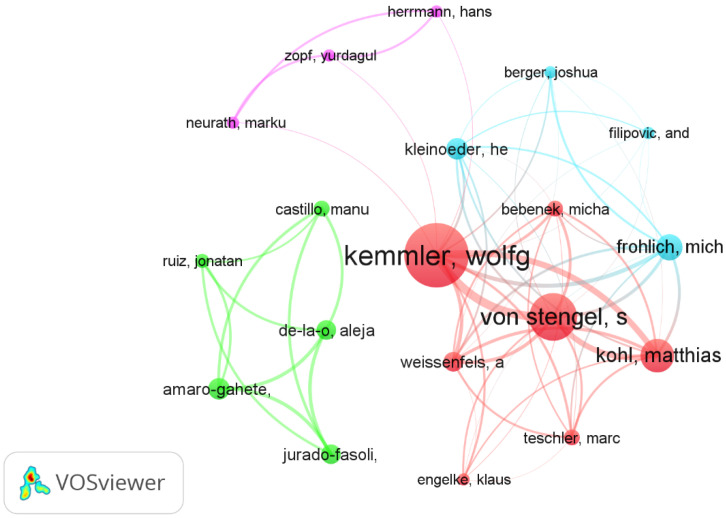
Co-authors graph on WB-EMS (19 prolific authors).

Figure 3 shows the graph of interrelationships between the 19 prolific authors, forming four clusters, obtained with VoSviewer software (1.6.18, Center for Science and Technology Studies, Leiden, Netherlands) using a normalization analysis with a fractionalization method (attraction: 10; repulsion: −4). Cluster 1 (Red) was the prolific, led by Kemmler (36 articles, accompanied by: Von Stengel (27 articles), Kohl (19 articles), Weissaefels (11 articles), Bebenek (9 articles), Teschler (9 articles) and Engelke (7 articles).

**Figure 4:**
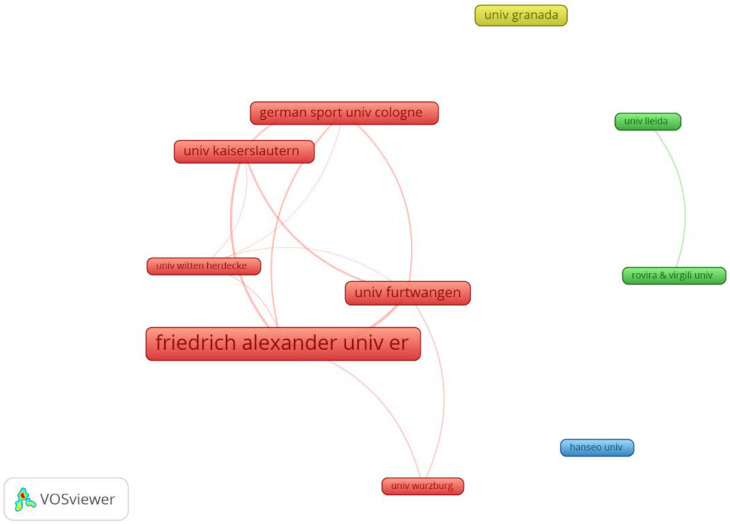
Graph of prolific organizational co-authors on WB-EMS.

A total of 113 organizations were found publishing on the target topic of this study. Only 10 of them presented three or more articles, with 23 organizations publishing two or more. The organization with the most publications was Friedrich Alexander University Erlangen Nurnberg (41 articles), forming a very prolific cluster with other German universities, as shown in Figure 4 with the interactions between the 113 organizations and the highest number of publications.

**Figure 5:**
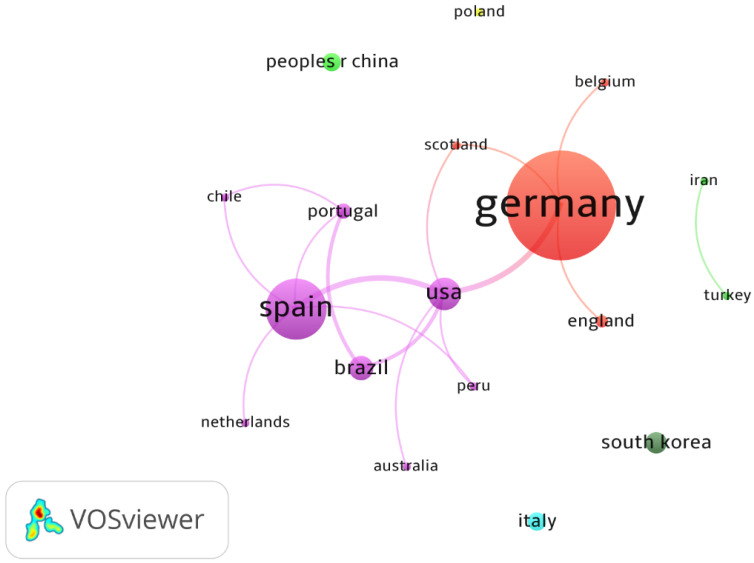
Countries/regions co-authored graphs on WB-EMS.

Germany (56 articles) and Spain (23 articles) were the countries that concentrated more than 75% of the publications on WB-EMS. Germany presented the highest production on the topic, with numerous universities involved, as can be seen in Figure 4. Figure 5 shows the 18 countries that contributed articles to the topic by author affiliation (Methods: Fractionization; Attraction: 8; Repulsion: 0). Two main clusters were found, led by Germany (Scotland, Belgium, and England) and Spain (USA, Brazil, Portugal, Chile, Peru, Australia and Netherlands).

Applying the Hirsch index (h-index) to select the articles with the highest number of citations, 20 papers were found to be cited 20 or more times (Table 3).

**Figure 6:**
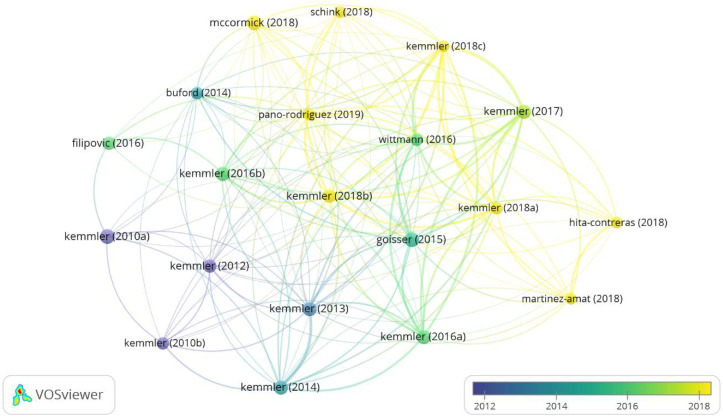
Graph with most relevant articles, according to h-index.

The most cited article was “Sarcopenic obesity and complex interventions with nutrition and exercise in community-dwelling older persons—a narrative review” [32] (Gossier, 2015) with 82 citations, followed by one of the pioneering articles on the topic: “Effects of whole-body electromyostimulation on resting metabolic rate, body composition, and maximum strength in postmenopausal women: the training and electrostimulation trial” [33] (Kemmel, 2010), with 72 citations. Figure 6 shows the graph with the 20 most relevant publications, according to the h-index.

**Figure 7:**
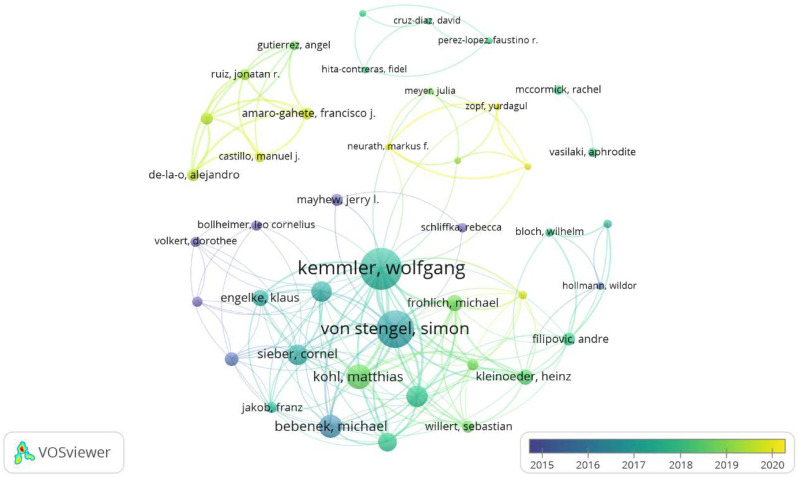
Author with more citations on WB-EMS.

Similarly, the 42 authors who presented at least 42 citations were selected and were considered the most prominent authors. Kemmler (776 citations) and Von Stongel (648 citations) were the most cited authors (Figure 7).

**Figure 8:**
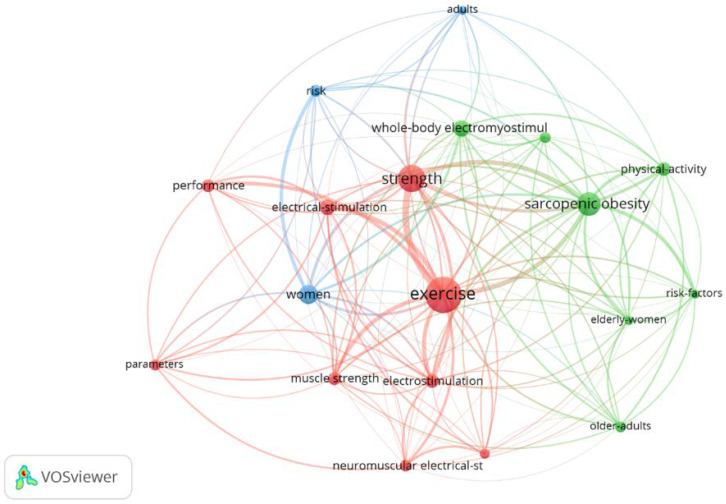
Graph with the most repeated keywords plus on WB-EMS.

Upon the analysis of the keywords plus found 336 words, the most relevant keywords were considered as the 19 most used by applying Zipf’s law. These words appeared 10 or more times in the articles that composed the complete data set. Four clusters were found with the relationships of these words; Figure 8 shows the configuration of these clusters and interrelationships between the 19 keywords with the most occurrences.

## 4. Discussion

This bibliometric analyzed the properties of 102 publication included in a citation index of WB-EMS studies conducted since 2010 (over the past 12 year). During this period, the trend of annual publications showed an exponential growth in publications, except in 2017, where there were only 2 publications. In 2021, the number of publications dropped, which may reflect the COVID-19 pandemic (Figure 1).

Figure 3, through a visual analysis of the distribution of countries, shows that Germany and Spain are the leading countries where most of the studies are being conducted. Additionally, there is some collaboration between countries. Research teams in the United States mainly collaborated with Germany, Spain and Brazil. The two countries that publish the most do not collaborate with each other. On the one hand, German researchers collaborate more with Belgium, Scotland and England and, on the other hand, the Spanish ones publish more with the Netherlands, Chile, Portugal and Peru.

The most prolific, influential and cited author in WB-EMS is Wolfgang Kemmler, followed by Simon Von Stengel (Table 2) with a wide network of connections and collaborations with other authors (Figure 2 and Figure 5). Moreover, in terms of the number of citations “Sarcopenic obesity and complex interventions with nutrition and exercise in community-dwelling older persons—a narrative review” [32] is the most cited article (Table 3). This study is a narrative review of the nutritional and exercise interventions for sarcopenic obesity in which WB-EMS is pointed out as time-saving option for positively impacting body composition and functional capacity. According the H-index (Figure 5), Wolgang Kemmler is the author of five of the six most relevant articles [1,34,36,59,69], in which the most relevant is the article by Sabine Goisser [32]. Interesting to note that the relevant articles are interventions made in special cohorts (sarcopenic obesity, postmenopausal woman and elderly).

As shown in Figure 2, most authors (75%) only have one published article. On the other hand, there is an author who is involved in about 35% of published articles. *Frontiers in Physiology* (23 articles), *Clinical Interventions in Aging* [6] and *International Journal of Environmental Research and Public Health* [6] are the journals where authors publish the most. On the one hand, *Frontiers in Physiology* is one of the largest journals in the world, covering more than 900 academic disciplines. On the other hand, the other two scientific journals are more related to aging and public health, both fields of application of this methodology.

From keyword analysis, the current research focuses on exercise, strength and sarcopenic obesity. Physical exercise is at the base of the application of WB-EMS, since the methodology is active, and superimposed on exercise. Strength development is one of the effects of using this methodology, which has already reported in several studios and in a recent meta-analysis [37]. On the other hand, the use of WB-EMS in people with sarcopenic obesity has been shown to be very effective, as we have previously mentioned.

The 17 references identified in this study are about the use and effects of the WB-EMS. To the best of our knowledge this bibliometric analysis is the first that provides a general a broad overview of the state of research on WB-EMS. Identifying the main authors, articles and most relevant institutions is extremely important for future researchers to be able to identify which lines of investigation have the most evidence and those that still need further investigation. Furthermore, it can help in future collaborations between new authors and more experienced authors for increasing and better research in the future.

There are two main limitations that we would like to point out in this study. First, the short time of study on WB-EMS; this type of study may have more meaning when there is more research time and number of studies. Second, the literature search was limited to Web of Science Core Collection databases, which might have resulted in election bias to the outcomes.

Other authors can utilize the results of this work to best know the WB-EMS topic and working in new hypotheses. The findings and their implications should be discussed in the broadest context possible. Future research directions may also be highlighted.

As for future research challenges, it is necessary to complement the data obtained in this manuscript with a systematic review and meta-analysis, and it would also be very interesting to perform bibliometric and systematic review analyses and specific meta-analyses in the different areas identified as lines of research on BM-EMS (exercise, strength and sarcopenic obesity).

## 5. Conclusions

This bibliometric analysis provides an overview of research findings into WB-EMS worldwide. There is an exponential growth trend in WB-EMS research since the first article published in 2010. Germany is the country that produces the most scientific knowledge on this topic (more than 75% of the articles). The leading institution is the Friedrich Alexander University Erlangen Nurnberg affiliation of the most relevant author Wolfgang Kemmler. In addition, *Frontiers in Psychology* is the most attractive journal for WB-EM researchers.

## Figures and Tables

**Table 1:** Core and zone 1 (Bradford’s zones) journals, according to publications for WB-EMS.

Bradford’s Zones	Journals	Quartile	Articles	% Articles	% O. Acc. Articles
**Core**	*Frontiers in physiology*	Q1	23	23%	23%
	*Clinical interventions in aging*	Q2	6	6%	28%
	*International journal of environmental research and public health*	Q1	6	6%	34%
**Zone 1**	*Journal of sports science and medicine*	Q2	3	3%	37%
	*Journal of strength and conditioning research*	Q2	3	3%	40%
	*Nutrients*	Q1	3	3%	43%
	*Deutsche zeitschrift fur sportmedizin*	Not Applicable	2	2%	45%
	*Evidence based complementary and alternative Medicine*	Q2	2	2%	47%
	*International journal of sports medicine*	Q2	2	2%	49%
	*Isokinetics and exercise science*	Q4	2	2%	51%
	*Maturitas*	Q1	2	2%	53%
	*Medicina-Lithuania*	Not Applicable	2	2%	55%
	*PloS One*	Q2	2	2%	57%

**Table 2:** Co-author prolific on WB-EMS.

Author	Main Affiliation	Country	Articles	Citations
1. Kemmler, W.	University of Erlangen Nurnberg	Germany	36	772
2. Von stengel, S.	University of Erlangen Nurnberg	Germany	27	648
3. Kohl, M.	University of Erlangen Nurnberg. University of Kaiserslautern	Germany	19	333
4. Frohlich, M.	University of Kaiserslautern	Germany	15	181
5. Amaro-Gahete, F.	University of Granada	Spain	12	113
6. Kleinoeder, H.	German Sport University Cologne	Germany	12	165
7. De-la-O, A.	University of Granada	Spain	11	113
8. Jurado-Fasoli, L.	University of Granada	Spain	11	113
9. Weissenfels, A.	University of Erlangen Nurnberg	Germany	11	270
10. Bebenek, M.	University of Erlangen Nurnberg	Germany	9	297
11. Castillo, M.	University of Granada	Spain	9	84
12. Teschler, M.	University of Erlangen Nurnberg	Germany	9	213
13. Ruiz, J.	University of Granada	Spain	8	94
14. Berger, J.	University of Kaiserslautern	Germany	7	62
15. Engelke, K.	University of Erlangen Nurnberg	Germany	7	170
16. Filipovic, A.	German Sport University Cologne	Germany	7	115
17. Herrmann, H.	Universiy of Erlangen Nurnberg	Germany	7	49
18. Neurath, M.	Universiy of Erlangen Nurnberg	Germany	7	49
19. Zopf, Y.	Universiy of Erlangen Nurnberg	Germany	7	49

**Table 3:** Articles with more relevance, according to h-index.

Article	Journal	Author	Year	Citations
Sarcopenic obesity and complex interventions with nutrition and exercise in community-dwelling older persons—a narrative review	*Clin Interv Aging*	Goisser [32]	2015	82
Effects of whole-body electromyostimulation on resting metabolic rate, body composition, and maximum strength in postmenopausal women: the training and electrostimulation trial	*J Strength Cond Res*	Kemmler * [33]	2010	73
Age-related changes in skeletal muscle: changes to lifestyle as a therapy	*Biogerontology*	McCormick [57]	2018	71
Impact of whole-body electromyostimulation on body composition in elderly women at risk for sarcopenia: the Training and ElectroStimulation Trial [TEST-III]	*Age*	Kemmler * [34]	2014	63
Whole-body electromyostimulation to fight sarcopenic obesity in community-dwelling older women at risk. Results of the randomized controlled FORMOsA-sarcopenic obesity study	*Osteoporosis Int*	Kemmler * [58]	2016	59
Effects of Whole-Body Electromyostimulation versus High-Intensity Resistance Exercise on Body Composition and Strength: A Randomized Controlled Study	*Evid-based Compl Alt*	Kemmler * [36]	2016	57
Whole-body electromyostimulation as a means to impact muscle mass and abdominal body fat in lean, sedentary, older female adults: subanalysis of the TEST-III trial	*Clin Interv Aging*	Kemmler * [16]	2013	55
Whole-body electromyostimulation and protein supplementation favorably affect sarcopenic obesity in community-dwelling older men at risk: the randomized controlled FranSO study	*Clin Interv Aging*	Kemmler * [59]	2017	54
Efficacy and Safety of Low Frequency Whole-Body Electromyostimulation [WB-EMS] to Improve Health-Related Outcomes in Non-athletic Adults. A Systematic Review	*Front Physiol*	Kemmler * [60]	2018	42
Effect of whole-body electromyostimulation on energy expenditure during exercise	*J Strength Cond Res*	Kemmler * [61]	2012	42
Effects of a Whole-Body Electrostimulation Program on Strength, Sprinting, Jumping, and Kicking Capacity in Elite Soccer Players	*J Sport Sci Med*	Filipovic * [38]	2016	37
Impact of whole body electromyostimulation on cardiometabolic risk factors in older women with sarcopenic obesity: the randomized controlled FORMOsA-sarcopenic obesity study	*Clin Interv Aging*	Whittman [62]	2016	29
Effects of Combined Whole-Body Electromyostimulation and Protein Supplementation on Local and Overall Muscle/Fat Distribution in Older Men with Sarcopenic Obesity: The Randomized Controlled Franconia Sarcopenic Obesity [FranSO] Study	*Calcified Tissue Int*	Kemmler * [63]	2018	28
Optimizing the Benefits of Exercise on Physical Function in Older Adults	*PM&R*	Buford [64]	2014	28
Effects of Whole-Body-Electromyostimulation on Body Composition and Cardiac Risk Factors in Elderly Men with the Metabolic Syndrome. The TEST-II Study	*Deut Z Sportmed*	Kemmler * [1]	2010	28
Effect of exercise alone or combined with dietary supplements on anthropometric and physical performance measures in community-dwelling elderly people with sarcopenic obesity: A meta-analysis of randomized controlled trials	*Maturitas*	Hita-Contreras [65]	2018	26
Effects of whole-body electromyostimulation combined with individualized nutritional support on body composition in patients with advanced cancer: a controlled pilot trial	*BMC Cancer*	Schink [66]	2018	25
Effect of whole-body electromyostimulation and/or protein supplementation on obesity and cardiometabolic risk in older men with sarcopenic obesity: the randomized controlled FranSO trial	*BMC Geriatr*	Kemmler * [63]	2018	24
Effects of whole-body ELECTROMYOSTIMULATION on health and performance: a systematic review	*BMC Complem Altern M*	Pano-Rodriguez [67]	2019	21
Exercise alone or combined with dietary supplements for sarcopenic obesity in community-dwelling older people: A systematic review of randomized controlled trials	*Maturitas*	Martinez-Amat [68]	2018	21

* It coincides with the prolific.

## Data Availability

The raw data supporting the conclusions of this article will be made available by the authors.

## References

[1] Kemmler W., Birlauf A., Von Stengel S. (2010). Effects of Whole-Body-Electromyostimulation on body composition and cardiac risk factors in elderly men with the metabolic syndrome. The Test-II study. Dtsch. Z. Sportmed..

[2] Von Stengel S., Bebenek M., Engelke K., Kemmler W. (2015). Whole-Body Electromyostimulation to Fight Osteopenia in Elderly Females: The Randomized Controlled Training and Electrostimulation Trial (TEST-III). J. Osteoporos..

[3] Kemmler W., Froehlich M., Von Stengel S., Kleinöder H. (2016). Whole-Body Electromyostimulation—The Need for Common Sense! Rationale and Guideline for a Safe and Effective Training. Dtsch. Z. Sportmed..

[4] Schuhbeck E., Birkenmaier C., Schulte-Göcking H., Pronnet A., Jansson V., Wegener B. (2019). The Influence of WB-EMS-Training on the Performance of Ice Hockey Players of Different Competitive Status. Front. Physiol..

[5] Wirtz N., Dörmann U., Micke F., Filipovic A., Kleinöder H., Donath L. (2019). Effects of Whole-Body Electromyostimulation on Strength-, Sprint-, and Jump Performance in Moderately Trained Young Adults: A Mini-Meta-Analysis of Five Homogenous RCTs of Our Work Group. Front. Physiol..

[6] Wirtz N., Filipovic A., Gehlert S., de Marées M., Schiffer T., Bloch W., Donath L. (2020). Seven Weeks of Jump Training with Superimposed Whole-Body Electromyostimulation Does Not Affect the Physiological and Cellular Parameters of Endurance Performance in Amateur Soccer Players. Int. J. Environ. Res. Public Health.

[7] Weissenfels A., Wirtz N., Dörmann U., Kleinöder H., Donath L., Kohl M., Fröhlich M., von Stengel S., Kemmler W. (2019). Comparison of Whole-Body Electromyostimulation versus Recognized Back-Strengthening Exercise Training on Chronic Nonspecific Low Back Pain: A Randomized Controlled Study. BioMed Res. Int..

[8] Choi G., Hyon P., Song J.E. (2016). Effects of the Micro-Training with EMS Device on Body Composition, Isokinetic Muscular Function, and Physical Fitness of Healthy 20’s Males. Korean Soc. Sports Sci..

[9] Schink K., Herrmann H.J., Schwappacher R., Orlemann T., Meyer J., Waldmann E., Wullich B., Kahlmeyer A., Fietkau R., Lubgan D. (2018). Whole-Body Electromyostimulation combined with personalized Nutritional Support improves the Body Composition of Patients with advanced Cancer. Internist.

[10] Niels T., Kersten J., Tomanek A., Baumann F. (2020). Pilot Case-Series: Can Short-Term WB-EMS be Effective in Cancer Patients?. Oncol. Res. Treat..

[11] Ricci P.A., Di Thommazo-Luporini L., Jürgensen S.P., André L.D., Haddad G.F., Arena R., Borghi-Silva A. (2020). Effects of Whole-Body Electromyostimulation Associated with Dynamic Exercise on Functional Capacity and Heart Rate Variability After Bariatric Surgery: A Randomized, Double-Blind, and Sham-Controlled Trial. Obes. Surg..

[12] Chisari E., Pavone V., Sessa G., Ravalli S., Musumeci G. (2019). Electromyostimulation and whole-body vibration effects in elder sarcopenic patients. Muscles Ligaments Tendons J..

[13] Filipovic A., Kleinöder H., Dörmann U., Mester J. (2011). Electromyostimulation—A Systematic Review of the Influence of Training Regimens and Stimulation Parameters on Effectiveness in Electromyostimulation Training of Selected Strength Parameters. J. Strength Cond. Res..

[14] Filipovic A., Kleinöder H., Dörmann U., Mester J. (2012). Electromyostimulation—A Systematic Review of the Effects of Different Electromyostimulation Methods on Selected Strength Parameters in Trained and Elite Athletes. J. Strength Cond. Res..

[15] Paillard T. (2018). Training Based on Electrical Stimulation Superimposed Onto Voluntary Contraction Would be Relevant Only as Part of Submaximal Contractions in Healthy Subjects. Front. Physiol..

[16] Kemmler W., von Stengel S. (2013). Whole-body electromyostimulation as a means to impact muscle mass and abdominal body fat in lean, sedentary, older female adults: Subanalysis of the TEST-III trial. Clin. Interv. Aging.

[17] Peterson M.D., Sen A., Gordon P.M. (2011). Influence of Resistance Exercise on Lean Body Mass in Aging Adults: A Meta-Analysis. Med. Sci. Sports Exerc..

[18] Pano-Rodriguez A., Beltran-Garrido J.V., Hernandez-Gonzalez V., Nasarre-Nacenta N., Reverter-Masia J. (2020). Impact of Whole Body Electromyostimulation on Velocity, Power and Body Composition in Postmenopausal Women: A Randomized Controlled Trial. Int. J. Environ. Res. Public Health.

[19] Jose Amaro-Gahete F., De la O., Alejandro, Robles-Gonzalez L., Joaquin Castillo M., Gutierrez A. (2018). Impact of two whole-body electromyostimulation training modalities on body composition in recreational runners during endurance training cessation. Ricyde Rev. Int. Cienc. Deporte.

[20] Kemmler W., Teschler M., Weissenfels A., Froehlich M., Kohl M., Von Stengel S. (2015). Whole-body electromyostimulation versus high intensity (Resistance exercise) training—Impact on body composition and strength. Dtsch. Z. Sportmed..

[21] Weissenfels A., Teschler M., Willert S., Hettchen M., Fröhlich M., Kleinöder H., Kohl M., von Stengel S., Kemmler W. (2018). Effects of whole-body electromyostimulation on chronic nonspecific low back pain in adults: A randomized controlled study. J. Pain Res..

[22] Konrad K.L., Baeyens J.-P., Birkenmaier C., Ranker A.H., Widmann J., Leukert J., Wenisch L., Kraft E., Jansson V., Wegener B. (2020). The effects of whole-body electromyostimulation (WB-EMS) in comparison to a multimodal treatment concept in patients with non-specific chronic back pain—A prospective clinical intervention study. PLoS ONE.

[23] Berger J., Ludwig O., Becker S., Kemmler W., Fröhlich M. (2021). Effects of an 8-Week Whole-Body Electromyostimulation Training on Cycling Performance, Back Pain, and Posture of a 17-Year-Old Road Cyclist. Int. J. Athl. Ther. Train..

[24] D’ottavio S., Briotti G., Rosazza C., Partipilo F., Silvestri A., Calabrese C., Bernardini A., Gabrielli P.R., Ruscello B. (2019). Effects of Two Modalities of Whole-body Electrostimulation Programs and Resistance Circuit Training on Strength and Power. Int. J. Sports Med..

[25] Amaro-Gahete F.J., De-La-O A., Sanchez-Delgado G., Robles-Gonzalez L., Jurado-Fasoli L., Ruiz J.R., Gutierrez A. (2018). Whole-Body Electromyostimulation Improves Performance-Related Parameters in Runners. Front. Physiol..

[26] Micke F., Kleinöder H., Dörmann U., Wirtz N., Donath L. (2018). Effects of an Eight-Week Superimposed Submaximal Dynamic Whole-Body Electromyostimulation Training on Strength and Power Parameters of the Leg Muscles: A Randomized Controlled Intervention Study. Front. Physiol..

[27] Berger J., Becker S., Ludwig O., Kemmler W., Frohlich M. (2020). Whole-body electromyostimulation in physical therapy: Do gender, skinfold thickness or body composition influence maximum intensity tolerance?. J. Phys. Ther. Sci..

[28] Pano-Rodriguez A., Beltran-Garrido J.V., Hernandez-Gonzalez V., Reverter-Masia J. (2020). Effects of Whole-Body Electromyostimulation on Physical Fitness in Postmenopausal Women: A Randomized Controlled Trial. Sensors.

[29] Ludwig O., Berger J., Becker S., Kemmler W., Fröhlich M. (2019). The Impact of Whole-Body Electromyostimulation on Body Posture and Trunk Muscle Strength in Untrained Persons. Front. Physiol..

[30] Park H.-K., Na S.M., Choi S.-L., Seon J.-K., Do W.-H. (2021). Physiological Effect of Exercise Training with Whole Body Electric Muscle Stimulation Suit on Strength and Balance in Young Women: A Randomized Controlled Trial. Chonnam Med. J..

[31] Kim J., Jee Y. (2020). EMS-effect of Exercises with Music on Fatness and Biomarkers of Obese Elderly Women. Medicina.

[32] Freiberger E., Goisser S., Porzel S., Volkert D., Kemmler W., Sieber C., Bollheimer C. (2015). Sarcopenic obesity and complex interventions with nutrition and exercise in community-dwelling older persons—A narrative review. Clin. Interv. Aging.

[33] Kemmler W., Schliffka R., Mayhew J.L., von Stengel S. (2010). Effects of Whole-Body Electromyostimulation on Resting Metabolic Rate, Body Composition, and Maximum Strength in Postmenopausal Women: The Training and ElectroStimulation Trial. J. Strength Cond. Res..

[34] Kemmler W., Bebenek M., Engelke K., Von Stengel S. (2014). Impact of whole-body electromyostimulation on body composition in elderly women at risk for sarcopenia: The Training and ElectroStimulation Trial (TEST-III). Age.

[35] Kemmler W., Engelke K., von Stengel S., Jakob F., Sieber C. (2016). Whole Body Electromyostimulation and Protein to Fight Sarcopenic Obesity in Women 70 Years and Older: Preliminary Data of the Formosastudy. Osteoporos. Int..

[36] Kemmler W., Teschler M., Weißenfels A., Bebenek M., Fröhlich M., Kohl M., von Stengel S. (2016). Effects of Whole-Body Electromyostimulation versus High-Intensity Resistance Exercise on Body Composition and Strength: A Randomized Controlled Study. Evid.-Based Complement. Altern. Med..

[37] Kemmler W., Shojaa M., Steele J., Berger J., Fröhlich M., Schoene D., von Stengel S., Kleinöder H., Kohl M. (2021). Efficacy of Whole-Body Electromyostimulation (WB-EMS) on Body Composition and Muscle Strength in Non-athletic Adults. A Systematic Review and Meta-Analysis. Front. Physiol..

[38] Filipovic A., Grau M., Kleinöder H., Zimmer P., Hollmann W., Bloch W. (2016). Effects of a Whole-Body Electrostimulation Program on Strength, Sprinting, Jumping, and Kicking Capacity in Elite Soccer Players. J. Sports Sci. Med..

[39] Evangelista A.L., Teixeira C.V.L., Barros B.M., de Azevedo J.B., Paunksnis M.R.R., de Souza C.R., Wadhi T., Rica R.L., Braz T.V., Bocalini D.S. (2019). Does whole-body electrical muscle stimulation combined with strength training promote morphofunctional alterations?. Clinics.

[40] Evangelista A.L., Pozzi M.L.B., Santos L.M., Barros B.M., de Souza C.R., Reis V.M., Bocalini D.S. (2021). Energy Expenditure in Hiit Whole Body Associated with Electromyostimulation. Rev. Bras. Med. Esporte.

[41] Jurado-Fasoli L., Amaro-Gahete F.J., De-La-O A., Castillo M.J. (2020). Impact of different exercise training modalities on energy and nutrient intake and food consumption in sedentary middle-aged adults: A randomised controlled trial. J. Hum. Nutr. Diet..

[42] Science CWo. http://www.webofknowledge.com/.

[43] Mongeon P., Paul-Hus A. (2016). The journal coverage of Web of Science and Scopus: A comparative analysis. Scientometrics.

[44] Harzing A.-W., Alakangas S. (2016). Google Scholar, Scopus and the Web of Science: A longitudinal and cross-disciplinary comparison. Scientometrics.

[45] Falagas M.E., Pitsouni E.I., Malietzis G., Pappas G. (2008). Comparison of PubMed, Scopus, Web of Science, and Google Scholar: Strengths and weaknesses. FASEB J..

[46] Chadegani A.A., Salehi H., Yunus M.M., Farhadi H., Fooladi M., Farhadi M., Ebrahim N.A. (2013). A comparison between two main academic literature collections: Web of Science and Scopus databases. arXiv.

[47] Bakkalbasi N., Bauer K., Glover J., Wang L. (2006). Three options for citation tracking: Google Scholar, Scopus and Web of Science. Biomed. Digit. Libr..

[48] Price D.D.S. (1976). A general theory of bibliometric and other cumulative advantage processes. J. Am. Soc. Inf. Sci..

[49] Dobrov G.M., Randolph R.H., Rauch W.D. (1979). New options for team research via international computer networks. Scientometrics.

[50] Bulick S. (1978). Book Use as a Bradford-Zipf Phenomenon. Coll. Res. Libr..

[51] Morse P.M., Leimkuhler F.F. (1979). Technical Note—Exact Solution for the Bradford Distribution and Its Use in Modeling Informational Data. Oper. Res..

[52] Coile R.C. (1977). Lotka’s frequency distribution of scientific productivity. J. Am. Soc. Inf. Sci..

[53] Hirsch J.E. (2005). An index to quantify an individual’s scientific research output. Proc. Natl. Acad. Sci. USA.

[54] Zipf G. (1932). Selected Studies of the Principle of Relative Frequency in Language.

[55] Waltman L., van Eck N.J., Noyons E.C.M. (2010). A unified approach to mapping and clustering of bibliometric networks. J. Informetr..

[56] Perianes-Rodriguez A., Waltman L., van Eck N.J. (2016). Constructing bibliometric networks: A comparison between full and fractional counting. J. Inf..

[57] McCormick R., Vasilaki A. (2018). Age-related changes in skeletal muscle: Changes to life-style as a therapy. Biogerontology.

[58] Kemmler W., Teschler M., Weissenfels A., Bebenek M., Von Stengel S., Kohl M., Freiberger E., Goisser S., Jakob F., Sieber C.C. (2016). Whole-body electromyostimulation to fight sarcopenic obesity in community-dwelling older women at risk. Results of the randomized controlled FORMOsA-sarcopenic obesity study. Osteoporos. Int..

[59] Kemmler W., Weissenfels A., Teschler M., Willert S., Bebenek M., Shojaa M., Kohl M., Freiberger E., Sieber C., von Stengel S. (2017). Whole-body electromyostimulation and protein supplementation favorably affect sarcopenic obesity in community-dwelling older men at risk: The randomized controlled FranSO study. Clin. Interv. Aging.

[60] Kemmler W., Weissenfels A., Willert S., Shojaa M., Von Stengel S., Filipovic A., Kleinöder H., Berger J., Fröhlich M. (2018). Efficacy and Safety of Low Frequency Whole-Body Electromyostimulation (WB-EMS) to Improve Health-Related Outcomes in Non-athletic Adults. A Systematic Review. Front. Physiol..

[61] Kemmler W., Von Stengel S., Schwarz J., Mayhew J.L. (2012). Effect of Whole-Body Electromyostimulation on Energy Expenditure During Exercise. J. Strength Cond. Res..

[62] Wittmann K., Sieber C., von Stengel S., Kohl M., Freiberger E., Jakob F., Lell M., Engelke K., Kemmler W. (2016). Impact of whole body electromyostimulation on cardiometabolic risk factors in older women with sarcopenic obesity: The randomized controlled FORMOsA-sarcopenic obesity study. Clin. Interv. Aging.

[63] Kemmler W., Grimm A., Bebenek M., Kohl M., von Stengel S. (2018). Effects of Combined Whole-Body Electromyostimulation and Protein Supplementation on Local and Overall Muscle/Fat Distribution in Older Men with Sarcopenic Obesity: The Randomized Controlled Franconia Sarcopenic Obesity (FranSO) Study. Calcif. Tissue Res..

[64] Buford T.W., Anton S.D., Clark D.J., Ba T.J.H., Cooke M.B. (2015). Optimizing the Benefits of Exercise on Physical Function in Older Adults. PMR.

[65] Hita-Contreras F., Bueno-Notivol J., Martínez-Amat A., Cruz-Díaz D., Hernandez A.V., Pérez-López F.R. (2018). Effect of exercise alone or combined with dietary supplements on anthropometric and physical performance measures in community-dwelling elderly people with sarcopenic obesity: A meta-analysis of randomized controlled trials. Maturitas.

[66] Schink K., Herrmann H.J., Schwappacher R., Meyer J., Orlemann T., Waldmann E., Wullich B., Kahlmeyer A., Fietkau R., Lubgan D. (2018). Effects of whole-body electromyostimulation combined with individualized nutritional support on body composition in patients with advanced cancer: A controlled pilot trial. BMC Cancer.

[67] Pano-Rodriguez A., Beltran-Garrido J.V., Hernández-González V., Reverter-Masia J. (2019). Effects of whole-body electromyostimulation on health and performance: A systematic review. BMC Complement. Altern. Med..

[68] Martínez-Amat A., Aibar-Almazán A., Fábrega-Cuadros R., Díaz D.C., García J.D.J., Pérez-López F.R., Achalandabaso A., Barranco-Zafra R., Hita-Contreras F. (2018). Exercise alone or combined with dietary supplements for sarcopenic obesity in community-dwelling older people: A systematic review of randomized controlled trials. Maturitas.

[69] Kemmler W., Von Stengel S., Teschler M., Weissenfels A., Bebenek M., Kohl M., Freiberger E., Bollheimer C., Goisser S., Sieber C. (2016). Whole-body electromyostimulation and sarcopenic obesity: Results of the randomized controlled FORMOsA-Sarcopenic Obesity Study. Osteologie.

